# Application of Stem Cell Therapy for ACL Graft Regeneration

**DOI:** 10.1155/2021/6641818

**Published:** 2021-08-02

**Authors:** Canlong Wang, Yejun Hu, Shichen Zhang, Dengfeng Ruan, Zizhan Huang, Peiwen He, Honglu Cai, Boon Chin Heng, Xiao Chen, Weiliang Shen

**Affiliations:** ^1^Department of Orthopedic Surgery, 2nd Affiliated Hospital, School of Medicine, Zhejiang University, Zhejiang 310009, China; ^2^Orthopaedics Research Institute of Zhejiang University, Zhejiang, China; ^3^China Orthopaedic Regenerative Medicine (CORMed), Hangzhou, China; ^4^Dr. Li Dak Sum and Yip Yio Chin Center for Stem Cell and Regenerative Medicine, Zhejiang University, Zhejiang 310000, China; ^5^Department of Sports Medicine, School of Medicine, Zhejiang University, Zhejiang 310000, China; ^6^Central Laboratory, Peking University School of Stomatology, Bejing 100081, China; ^7^Department of Orthopedics, Huzhou Central Hospital, Affiliated Central Hospital of Huzhou University, Zhejiang University Huzhou Hospital, Huzhou, Zhejiang 313000, China

## Abstract

Graft regeneration after anterior cruciate ligament (ACL) reconstruction surgery is a complex three-stage process, which usually takes a long duration and often results in fibrous scar tissue formation that exerts a detrimental impact on the patients' prognosis. Hence, as a regeneration technique, stem cell transplantation has attracted increasing attention. Several different stem cell types have been utilized in animal experiments, and almost all of these have shown good capacity in improving tendon-bone regeneration. Various differentiation inducers have been widely applied together with stem cells to enhance specific lineage differentiation, such as recombinant gene transfection, growth factors, and biomaterials. Among the various different types of stem cells, bone marrow-derived mesenchymal stem cells (BMSCs) have been investigated the most, while ligament stem progenitor cells (LDSCs) have demonstrated the best potential in generating tendon/ligament lineage cells. In the clinic, 4 relevant completed trials have been reported, but only one trial with BMSCs showed improved outcomes, while 5 relevant trials are still in progress. This review describes the process of ACL graft regeneration after implantation and summarizes the current application of stem cells from bench to bedside, as well as discusses future perspectives in this field.

## 1. Introduction

Anterior cruciate ligament (ACL) injuries account for more than 50% of all knee injuries [1], which may cause knee instability, resulting in meniscal damage and osteoarthritis. When tears occur, ACL reconstruction (ACLR) surgery is usually undertaken, which yields the best therapeutic effects and postoperative evaluation scores for patients [2]. There are over 175,000 patients undergoing ACL surgery annually in the US alone [3], but more than 10% of patients experience reinjuries, muscular atrophy, delay in healing, poor proprioception, and graft failure after reconstruction in long-term follow-ups [4–7]. Hence, the major challenge is how to improve postoperative graft healing.

After transplantation, the graft goes through a complex three-staged healing process involving necrosis, remodeling, and ligamentization, which may take around 2 years [8]. Moreover, fibrous scar tissue is often formed at the interface, instead of a natural insertion [9, 10]. When a tissue is characterized by poor healing capacity, such as tendon and ligament, regenerative strategies are usually considered. Several common regenerative approaches, such as stem cells, biomaterials, and bioactive molecules, have been investigated and proven to be effective [11–13]. Among these, stem cells are extremely appealing, due to their self-renewal capacity, long-term viability, and multilineage differentiation potential [14]. In particular, mesenchymal stem cell (MSC) can differentiate into various terminally differentiated lineages, which can be utilized to engineer mesenchymal-derived tissues, and also promote healing by secreting various immunoregulatory molecules, such as paracrine trophic mediators [15, 16]. To induce stem cells to differentiate into a specific lineage, various differentiation inducers are usually utilized, such as recombinant gene transfection, growth factors, and biomaterials. Indeed, an increasing number of preclinical research studies have confirmed that inducers could enhance bone-to-tendon healing with better biomechanical properties and more mature tissue formation. Several clinical trials have been attempted, but so far, it is still uncertain whether stem cell augmentation could facilitate the healing process.

The purpose of this review is to describe the natural healing process after ACL graft implantation and summarize the current application of stem cells from bench to bedside, as well as discuss future prospects in this field.

## 2. Process of ACL Graft Regeneration

From the posterior part of the inner surface of the lateral femoral condyle, the ACL runs anteriorly, medially, and distally to the tibia [17]. The main component of ACL tissue is constituted of parallel and closely arranged collagen fibers, and fibroblasts are distributed along the long axial among the collagen fibers [18]. There are three characteristic stages of graft healing after ACL reconstruction in both humans and animals [19]: (i) early phase associated with necrosis and hypocellularity, (ii) remodeling phase associated with revascularization and cell activities, and (iii) ligamentization phase associated with restructuring towards the native ACL [20].

**Figure 1:**
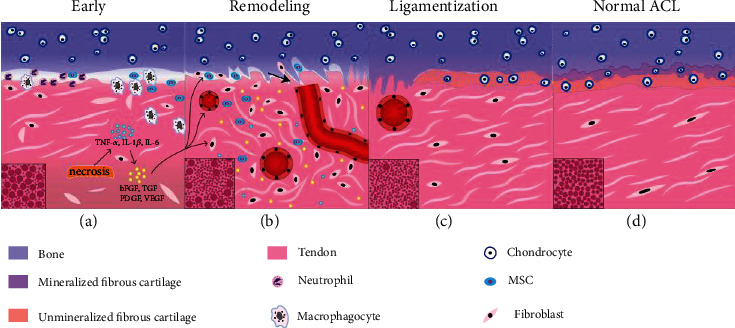
Schematic model for graft regeneration after ACL reconstruction. (a) Early stage characterized by necrosis, fiber disintegration, and cytokine release; neutrophils, macrophagocytes, and mesenchymal stem cells (MSC) can be observed in the interface in order, and then macrophagocytes and MSC migrate into the inner tendon. The collagen fibers displayed a bimodal distribution, with large ones constituting the majority; (b) remodeling stage marked with Sharpey fibers (arrow), cell migration, vascularization, ECM remodeling, various growth factor activities, and disordered organization of collagen fibers (bimodal distribution with small ones constituting the majority); (c) ligamentization stage marked with vascularization gradually disappearing, fibrocartilage formation, and ordered collagen with almost unimodal small fibers; (d) normal ACL, 4-layer direct insertion including ligament, fibrocartilage, mineralized fibrocartilage, and bone in order. The collagen fibers showed unclear bimodal distribution.

During the early stage, necrosis occurs in the graft centra, which leads to a release of various cytokines, such as tumor necrosis factor- (TNF-) *α*, interleukin (IL) 1-*β*, IL-6, and chemokines, which may trigger growth factor expression [21, 22]. Some host cells (neutrophils, macrophages, and MSC) migrate to the graft periphery [11, 12, 21], and towards the inner tendon [11]. Collagen fibrils begin disintegrating [13], and no graft revascularization could be observed [23, 24]. The collagen fibers of tendon display a bimodal distribution, with large collagen fibers constituting the majority. However, during healing, small fibers increase while large ones decrease (Figure 1(a)). Additionally, new surgery with attached graft may skip early necrosis, which retains the native blood supply [25, 26].

During the remodeling stage, large amounts of growth factors are released, which stimulates cell migration and proliferation as well as extracellular matrix synthesis and revascularization [22, 27, 28]. The hypercellular region at the perimeter consists of mesenchymal stem cells and fibroblasts [29]. Activated fibroblasts secrete various growth factors, which almost completely cease at the end of the remodeling stage [22]. The large diameter collagen fibrils get depleted [20], while the Sharpey-like fibers form to counteract shear stress and to attach the tendon graft to bone [30] (Figure 1(b)).

During the maturation stage, cellularity and mechanical properties become gradually similar to intact ACL but never reach the original levels [31, 32]. Progressive mineralization occurs, with subsequent bony ingrowth into the graft surface. Small collagen fibers predominate while large ones could hardly be seen, which differ significantly from normal ACL, with an unclear bimodal distribution (Figures 1(c) and 1(d)). Moreover, during this stage, more osteoarthritic changes and cartilage damage could be observed, with no significant differences in the expression of inflammatory cytokines or biomarkers [33].

Based on the above description, the graft healing process is slow and requires a long duration. The remodeling stage is finished by 9 months at the earliest [20, 34], and ligamentization could be observed after 2 years [8]. In the clinic, patients are usually recommended to return to low and moderate intensity exercise after 6 months [35–37], and typically regain about 85% function eventually [38]. Hence, a safe and effective approach to expedite the healing process is needed to restore the natural biomechanics of tendon, which is required for rapid return to preinjury activity levels.

## 3. Stem Cell Therapy for Graft Regeneration

**Figure 2:**
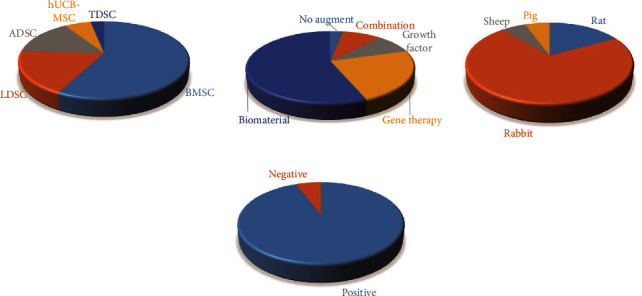
Features of included animal studies. (a) Cell resources; (b) augmentations; (c) animal models; (d) general study outcomes. BMSC: bone marrow-derived mesenchymal stem cells; ADSC: adipose-derived stem cells; hUCB-MSC: human umbilical cord blood-derived mesenchymal stem cells; TDSC: tendon-derived stem cells; LDSC: ligament-derived stem cells.

Stem cells show remarkable ability for self-renewal, long-term viability, and multilinear culture [14], which is an essential element in tissue engineering technology. In different cultures, stem cells could differentiate into nerve cells, hepatocytes, or blood cells. Combined with materials science, it is possible to construct similar tissues and organs to substitute the injured part. It has been widely proven that stem cells are effective in many diseases, such as central nervous system damage, and corneal destruction [39, 40]. Recent scientific literature has demonstrated promising outcomes of stem cell augmentation for ligament reconstruction in animal models [41–43] (Figure 2 and Table 1). However, the application of stem cells in ACLR requires further consideration of cell resource, differentiation induction, and cell fate.

### 3.1. Selection of Stem Cell Sources

There are several common cell sources in tissue engineering, such as embryonic stem cells (ESCs), induced pluripotent stem cell (iPSC), adipose tissue-derived stem cells (ADSCs), bone marrow-derived mesenchymal stem cells (BMSCs), and tendon/ligament stem/progenitor cells (TDSCs/LDSCs). In particular, MSC is the focus of much interest, as these cells are easily isolated from a variety of adult tissues and cultured *in vitro*. Cells from different sources have varying propensities to differentiate into various tendon/ligament lineages, and hence, it is imperative to weigh the pros and cons of various different stem cell types (Table 2).

#### 3.1.1. BMSC

BMSCs have multipotential capacity to differentiate into osteoblasts, chondrocytes, and adipocytes and hence have been most widely studied for enhancing tendon-bone healing, yielding satisfactory outcomes (Figure 2(d)). Sakaguchi compared the proliferative capacities of different stem cell types and observed that BMSCs were retained even at passage 10, whereas that of ADSCs was lost at passage 7 [44], thus showing the greater stability of BMSCs. However, these cells are not considered as the optimal choice due to the risk of ectopic ossification and donor injury. The therapeutic effects of BMSCs are thought to result from migration of the cells to inflammatory sites and suppression of inflammation. They are rarely involved in colonizing the healing tissue as part of the tissue repair mechanisms [45].

Lim et al. [46] implanted hamstring tendon autografts into the bone tunnel in rabbits, which was coated with MSCs embedded within a fibrin glue carrier in one limb, and fibrin glue only in the other limb, resulting in cartilage-like insertions rather than scar tissue. A similar study showed that BMSCs could decrease tunnel widening [47].

#### 3.1.2. ADSC

ADSCs have the advantages of abundant and ready availability, as well as capacity for secreting various factors, such as VEGF, hematopoietic factors, and immunoregulatory factors, to promote tissue repair and growth. Over 500 times more stem cells can be obtained from adipose tissue than from an equal tissue volume of bone marrow [48], and proteomic analysis of ASC secretome identified a total of 2416 distinct proteins [49]. In addition, ADSCs show lower risk of ectopic ossification, with less immunogenicity than BMSCs [50], causing less damage to the donor site, without the limitations associated with age-related decline of BMSCs. Indeed, ADSCs have demonstrated their suitability for various cell therapy applications including angiogenicity, osteogenicity, immunomodulation, and promotion of tissue remodeling [51, 52]. However, a study showed that ADSCs cannot continuously upregulate ligament-related markers with growth factors *in vitro*, as it exhibits a bias towards adipogenic differentiation [53].

It has been reported that ADSCs promote the early healing processes of tendon and bone in rabbits [43]. But Teuschl et al. [54] found that additional ADSCs did not result in any additional benefit for osteointegration, as compared with the silk scaffold group histologically, which showed ambiguous function.

#### 3.1.3. TDSC/LDSC

It has been reported that tissue-specific stem cells may retain a residual “epigenetic memory” of their tissue of origin [55]. When back at their tissue of origin, they could adapt to the environment better, survive longer, and differentiate more easily. TDSCs were first isolated from human hamstring tendon in 2007 [56], while a later study showed the possibility of isolating TDSCs from very small fragments of tendon tissue [57]. These cells proliferated faster, exhibited higher clonogenicity and less immunogenicity, and had more multilineage differentiation potential than BMSCs [58, 59]. However, the purity of TDSC populations is highly debatable, as it displayed lower adipogenic and osteogenic capacities than ADSCs [60], and lower multilineage differentiation potential than LDSCs [61]. TDSC-related studies are rare but seem promising, exhibiting high tenogenic potential and maintaining high chondroosteogenic gene expression [59].

Originating from the ligament tissue [62], CD90+CD73+ LDSCs tend to differentiate into ligament-committed cells or chondrocytes, as compared with BMSCs [63, 64]. The application of LDSCs *in vivo* has yielded generally positive results, when combined with silk scaffold, cell sheet, and injection [65, 66]. In particular, CD34+ vascular cells from ligament tissue are considered as another type of adult stem cell and have proven efficacious in tendon-bone regeneration [66, 67]. As a promising cell source, ACL-derived iPSCs are also under study [68]. The common problem of both is that low cell numbers necessitate expansion, which may influence phenotypic maintenance. Still, TDSCs and LDSCs are considered the most promising cell types for ACL regeneration.

#### 3.1.4. Other Stem Cell Types

hUCB-MSCs: hUCB-MSCs have the advantages of noninvasive isolation method, superior tropism, and high differentiation potential. Transplantation in rabbits enhanced bone-tendon healing effectively, without immune rejection [69, 70], while the application of human amniotic mesenchymal stem cells (hAMSCs) is still under research [71].

Synovium-derived MSCs (sMSCs): after injury, a local increase of MSCs was observed, and these MSCs were identified as sMSCs rather than BMSCs [72]. sMSCs can potentially promote collagen fiber production, which resembles Sharpey's fibers at the early stage.

ESC/iPSCs: ESCs could differentiate into any tissue or cell type, but therapeutic applications of these cells have been subjected to serious and prolonged legal/ethical discussion. On the other hand, iPSCs avoided ethical issues associated with ESC and also offered the possibility for autologous regeneration of any tissue. Cord and peripheral blood are attractive sources of reprogrammable cells for generating iPSCs [73, 74]. As a promising cell source, ACL-derived iPSCs are still under research [68]. But current outcomes of therapeutic applications in animal models seem controversial, with transplantation of ESCs into the knee joint of mice resulting in teratoma formation and subsequent destruction of the joint [75]. By contrast, composite grafts with iPSCs in pigs showed similar morphological and biochemical characteristics to normal ACL [76].

Exosome: no related research studies have been reported yet. However, the application of exosomes in tendon injury and tendinopathy in animal models showed satisfactory outcomes, which enhanced osseointegration, biomechanics, and histology [77–79], which is a promising therapeutic strategy for ACLR.

### 3.2. Differentiation Induction

#### 3.2.1. Biologic Factors

It is a consensus that growth factors could regulate cell proliferation, ECM elaboration, neovascularization, and mechanical properties. Hence, knowing the exact signaling mechanisms involved in ligament development and repair are essential for improving ACL regeneration, but our current knowledge is much limited and further research needs to be done. Functionally, it has been empirically shown that various growth factors exert positive effects on ligament tissues. Such as transforming growth factor (TGF), fibroblast growth factor (FGF), insulin-like growth factor (IGF), platelet-derived growth factor (PDGF), and epidermal growth factor (EGF), with all having been proven to increase cell proliferation, fibroblastic differentiation, and ECM deposition. As a combination of these factors, PRP could induce mass release of growth factors within one hour following intra-articular administration, which seems a convenient and efficient tool, but related meta-analysis studies found no significant benefit for ACLR in the clinic [80, 81].

Teng et al. found that PRP promoted BMSC osteodifferentiation *in vitro.* Moreover, PRP+BMSCs yielded better tendon-bone healing in rabbits [82]. Single growth factor, such as VEGF [83], also achieved good outcomes. To maintain the effects of these cytokines, gene therapy is a good solution. Runx2 gene upregulated the expression of osteogenic markers and enhanced tendon-bone healing with more new bone tissue formation, without heterotopic ossification [84]. The same results were achieved with BMP2, bFGF, TGF, VEGF, and PDGF gene transfection. Cotransfection of multiple genes is more powerful and efficient for osteogenic differentiation rather than either single gene therapy in Chen et al.'s study [85].

#### 3.2.2. Mechanics

Mechanical loading has been demonstrated to influence cell proliferation, differentiation, apoptosis, and ECM production without growth factors [86–88]. In fibroblasts, mechanical stimulus has been shown to increase cell proliferation, and ECM deposition [89]. It improves tendon-bone healing after ACLR by increasing the amount of fibrocartilage and mechanics. *In vitro*, BMSC/TC coculture stimulated by mechanical stretch showed higher expression levels of collagen I/III, alkaline phosphatase, osteopontin, and tenascin C [90], as well as BMSC alone [88]. In fact, the time, direction, magnitude, and frequency of mechanical stimulation would all influence the cell condition. Early mechanical loading on MSCs inhibited the expression of collagen type I, collagen type II, and fibronectin but enhanced these during the proliferation stage [91]. 8% but not 4% cyclical strain on ligament fibroblasts resulted in better proliferation and collagen production [92]. But it is difficult to control these mechanical parameters *in vivo*, so we need further investigations of the cultured environment before implantation. These could explain how prolonged immobilization would result in the mechanics of damage within the clinic [93].

#### 3.2.3. Biomaterials

In tissue engineering, cell differentiation can be induced by growing the cells on scaffolds with specific composition, architecture, and physicochemical and mechanical properties. Biomaterials not only play a load-bearing role in ACL reconstruction but is also a differentiation inducer.

In native ACL, type I collagen constitutes roughly 90% of the tissue volume, so the use of collagen-based scaffolds has been extensively investigated. Collagen could promote tenogenic differentiation induction, and the collagen-induced tenogenic cells could then arrest osteogenic differentiation mediated by paracrine signals [94]. But immunogenicity and low mechanical strength often limit the application of collagen-based scaffolds. Similar to collagen, silk is a natural biologic material with good tensile strength and biodegradation, but its limited cell adhesion requires some special modification, such as with arginine–glycine–aspartic acid. Silk scaffolds have also been shown to support BMSC attachment and proliferation within a three-dimensional environment and can induce synthesis of fibroblastic markers upon the application of dynamic mechanical loading [95]. Moreover, the hydrophilic properties of silk also influence the proliferation of seeded cells [96]. Electrospinning is a popular and simple technique for fabricating scaffolds with fiber diameters in the nanometer to micron range. Studies showed good capacity of polymer material-based electrospun fibers in promoting tendon fibroblast and MSC proliferation, as well as ECM deposition [97, 98]. Various mechanical parameters of different materials may affect the differentiation of stem cells, such as elastic modulus [99, 100], hydrophilicity or hydrophobicity [101], and substrate topography [102]. Stem cells seeded on aligned nanofibers displayed a more elongated shape with more Scx and ECM marker expression than randomly oriented nanofibers [102, 103]. Graphene-quantum dots could promote MSC osteogenic and adipogenic differentiation [104].

So far, silk scaffold, electrospun scaffold, and decellularized allograft with BMSCs have demonstrated good osseointegration capacity [105–107]. To simulate the insertion stratified structure better, a triphasic silk-based graft was established with BMSCs, chondrocytes, and osteoblasts seeded on different areas of the graft [42]. More novel materials combined with biologics are gaining in popularity.

### 3.3. Cell Fate

The fate of implanted stem cells remains controversial. Ju et al. used the fluorescent marker Dil dye to track implanted sMSCs, which initially stayed at the tendon-bone interface, and then differentiated into fibroblasts, with the potential of producing collagen fibers or secreting various cytokines for collagen fiber synthesis. But DiI-labeled cells could no longer be observed after 4 weeks [108]. There are three plausible reasons to explain this: missing label, cell replacement, or apoptosis. Lui et al. used the grafts wrapped with the GFP-TDSC sheet for ACLR, but only few GFP+ cells could be detected at the tunnel interface and the intra-articular graft midsubstance, with the cell number reducing with time [59]. Takeuchi et al. used engineered Tg pigs to track how endogenous cells infiltrate into the graft [12]. The graft was first surrounded by synovia-like tissue with fluorescence at first, then a large number of metabolically active oval cells infiltrated the peripheral region of the graft, resulting in a shift to an equal distribution of oval and spindle-shaped cells. Eventually, spindle-shaped fibroblast-like cells were uniformly distributed, resembling the natural ACL histology.

In some ACL injury models, exogenous cells were detected in the synovium, injured ACL, meniscus, cartilage of femoral condyles, and myotendinous junction of the quadriceps [109, 110]. Transplanted MSCs may produce growth factors such as PDGF, bFGF, and TGF-*β*, which promote native ACL cell proliferation and migration [111]. Maerz et al. found that tail-injected circulating MSCs preferentially migrate to the synovium of the injured joint, with the upregulation of SDF-1 (chemokines) in the synovial fluid. However, MSC did not enter the intra-articular tissues [110].

The objective of these studies was to form a normal insertion structure, but the source of newly formed fibrocartilage cells remains a mystery. Due to differentiation of the original cells, whether these are derived from transplanted exogenous stem cells or recruited endogenous cells remains ambiguous. How do these MSCs differentiate into fibroblast or other lineages? Tracking of cell fate needs to be more rigorously investigated.

## 4. Clinical Applications

**Figure 3:**
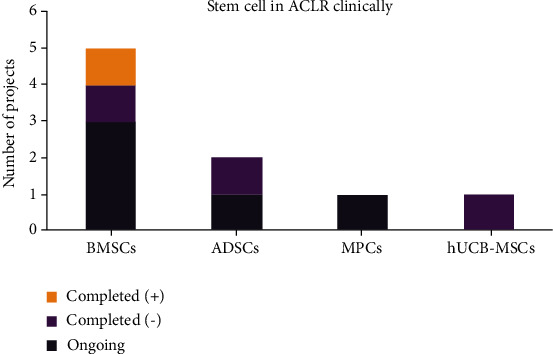
The number of ongoing and completed clinical projects with positive or negative results in the application of different stem cell lineages after ACL reconstruction. BMSC: bone marrow-derived mesenchymal stem cells; MPC: mesenchymal precursor cell; ADSC: adipose tissue-derived stem cell; hCDB-MSC: human cord blood-derived mesenchymal stem cell.

The search in electronic databases such as PubMed, Embase, and Cochrane Library resulted in 9 clinical trials (Figure 3), including 5 ongoing trials and 4 completed trials [112–115] (trial details see Appendix), highlighting the ongoing evolution of this field.

Based on the published results of completed clinical trials, the overall outcome was quite disappointing (Table 3). Wang et al. showed that the injection of allogeneic BMSCs after ACL reconstruction is safe and tolerable, improving the symptoms and delaying the progress of OA [112]. However, Silva et al. found no significant acceleration in tendon-bone healing with MRI [114]. Alentorn-Geli et al. utilized ADSCs in 20 soccer players with ACL reconstruction and found no statistically significant difference compared to ACLR alone, with respect to pain, biomechanical functions, and MRI scores [115]. Park et al. [116] conducted a 2-year follow-up with patients with hUCB-MSC augmentation and found no statistical differences in biomechanical functions, arthroscopic findings, or tunnel enlargement. Additionally, a noncontrolled trial utilizing autologous bone marrow aspirate combined with PRP and platelet lysate found safe outcomes with MRI and evaluation of clinical function [113].

## 5. Prospects

### 5.1. Cell Transplantation

Though preclinical studies have shown promising outcomes, the general clinical effects of stem cells on ACL graft regeneration are controversial, and the heterogeneity of transplanted stem cells needs to be further investigated with more high-quality research studies needed for an accurate and comprehensive conclusion. The clinical application of stem cells is a complex process, in view of the host tissue environment, time, cell adhesion, and dose. Due to the poor blood supply and insufficient nutrition provided by the articular cavity, too much cell injection will lead to necrosis, while too little will not yield a satisfactory effect. Dose- and time-dependent clinical research studies need to be carried out.

The timing of injecting or transplanting stem cells requires further consideration. Based on the process of graft regeneration as described above, two different therapeutic strategies for utilizing stem cells in ACL regeneration have been proposed: (a) during the early stage, transplanted stem cells make up for the hypocellularity, and the immunoregulatory property of MSCs (especially BMSCs) reduces inflammation reaction, as well as facilitate recruitment/activation of endogenous stem cells. Macrophages would accumulate for repair at the tendon-bone interface but often result in the formation of a scar tissue rather than normal insertion site [117], and so the regulation of macrophages by stem cells can enhance tendon-bone healing; (b) the application of stem cells during the remodeling stage may avoid apoptosis of transplanted cells due to poor blood supply. After angiogenesis and ECM deposition, the inner environment may be more suitable for stem cells to implant during the noninflammatory stage [118], but formed ECM may block the implanted cells to migrate towards the inner tendon. Additionally, abundant growth factors such as bFGF, TGF-*β*1, and PDGF [22] have demonstrated potent effects on tenogenic differentiation induction [119–121].

Different delivery methods have their own pros and cons. To attract stem cells into the scaffold, chemokines can be applied [65]. To simulate the insertion stratified structure, a triphasic silk-based graft was established with different cell types [42, 122], with specific induction treatment being applied to different parts of the graft. Decellularized allogenic scaffold is more similar to the original environment and enables easy seeding of cells [107]. To solve the problem of biocompatibility, biodegradability, and immunogenicity, cell sheet is a new option, which can be harvested from temperature-responsive culture dishes, and it has indeed shown promising outcomes in animal studies [59, 66]. In the clinic, due to the limitations of biomaterial approval, most trials deliver stem cell via injection, which often results in substantial loss of MSCs. Grafts wrapped in stem cell collagen seem a safe and simple solution. In summary, a carrier with great natural biodegradability, cell adhesion, biomechanics, biocompatibility, and insertion spatial simulation is required, and silk-based scaffolds have shown promising potential.

### 5.2. Differentiation of Stem Cells into Tendon/Ligament Cell Lineages

In embryos, tendon development requires both physiological and biomechanical stimulation [123, 124]. Temporal coordination of various physiological signals at early developmental stages, such as TGF-*β*, BMP, and FGF [125–127], as well as biomechanical stimulation at later stage [123], promotes tenogenic differentiation. The origin of ACL is still under research. Most joint tissues derive from GDF5(+) mesenchymal cell [128], of which Lgr5+/Scx+/Col22a1- interzone cells are restricted within the ligament lineage [129]. Scx+/Sox9+ precursors are also considered as the origin of the ACL [130], although exact signaling mechanisms involved in ligament development are still unclear. Several markers of embryonic tendon development were identified, but these do not provide functional properties. Based on embryonic tendon development, step induction is a logical method for simulating the development of tenocytes, with enhanced self-renewal, and long-term viability. Chen et al. induced hESCs to differentiate into MSCs and subsequently allow the MSCs to form tendon-like tissues with mechanical stress *in vitro* and *in vivo* [131].

Learning from embryonic tendon development can improve tendon tissue engineering strategies with adult stem cells, and tenogenic cues and markers will need to be established for step-wise induction [132]. Some studies have delivered MSCs together with exogenous proteinogenic growth factors to induce tenogenic differentiation. TGF is considered as an inducer of the tendon transcription factor Scx [70, 127], which can direct MSC differentiation towards the tenogenic lineage [119]. FGF mainly promotes matrix production for tendon maturation [120], and FGF4 treatment has been shown to significantly downregulate the gene expression levels of all tendon markers (Scx, TGF*β*2, Tnmd, Col I, and elastin) in MSCs but can only downregulate the mRNA levels of elastin in TDSC [121]. The BMP family of growth factors is essential to both osteogenic and chondrogenic differentiation [126], which may activate cytoskeletal reorganization or the Smad signaling pathway [133, 134]. In particular, BMP-12/13/14 signaling has been shown to be proteinogenic [135]. CTGF also plays an auxiliary role during tenogenic differentiation, by activating Scx, Tnmd, and other ECM marker expression, inducing fibroblastic effect and ECM production [136, 137]. In addition, Wnt signal was found to induce Tnmd expression in BMSCs via glycogen synthase kinase-3 [138]. These signaling factors play key roles in tendon differentiation and regeneration.

All stem cell therapies have the inherent risk of tumorigenicity, due to the aberration of chromosomal, copy number, and single nucleotide, hindering clinical translation [139, 140]. Hence, some researchers have turned to exosomes as an alternative, the specific vesicles secreted by stem cells, which can directly deliver the bioactive factors with low risk of tumorigenicity and undesired spontaneous differentiation. A similar tool is conditioned medium (CM), which represents a mixture of different factors secreted by the cells. The application of BMSC-CM accelerates graft-bone incorporation and midsubstance ligamentization and enhances differentiation as well [141]. These cell-free preparations have the advantages of less ossification, less calcification, and easy restoration, with various different proteins, nucleic acids, and lipid components being linked to their potency.

### 5.3. Current Challenges in Stem Cell Therapy for ACLR

Although challenges exist, preclinical evidence predicts a promising future for stem cell approach to ACLR, despite most (3/4) clinical research studies showing controversial outcomes. Currently, there are several ongoing human clinical trials in this area. Due to few studies on stem cell therapy for ACLR, we are unable to conduct a deep meta-analysis in this systematic review. In general, how exactly stem cells participate in human ACL regeneration and whether it has clinical benefits will require further study.

In addition, because stem cell transplantation is a biological therapeutic strategy, the stability and oncogenicity of stem cells require consistent long-term safety verification. The published scientific literature confirms the short-term (<24 months) safety and tolerance of stem cells in ACLR, but the implanted cells need long-term tracking, which has been poorly studied to date.

Third, the choice of the stem cell source is another important consideration. Stem cells derived from different sources all showed good capacity in promoting regeneration, but their relative effects need to be compared to optimize the therapeutic efficacy. With respect to availability and ease of isolation, ADSCs and BMSCs may have advantages over other stem cell types. In terms of proliferative capacity and ligamentous differentiation potential, TDSC/LDSC is regarded as having the most potential, but limited cell quantity may limit clinical applications. Proper differentiation of alternative stem cell lineages either *in vitro* or *in vivo* will be particularly crucial, because they are capable of differentiating into multiple tissue types. Current applications in humans are at the primary stage, so the differentiation induction *in vivo* is not mature and safe. Moreover, the implantation methodology and cell fate have been discussed previously, including the dose, time, supplementary agent, and material.

## 6. Conclusion

Almost all utilized stem cell lineages showed good capacity in promoting tendon-bone regeneration in animal models. Among the various different stem cell types, BMSCs are most commonly investigated, while LDSC/TDCS showed better potential for tendon/ligament lineage-specific differentiation. With differentiation inducers, such as growth factors, mechanical stimuli, and biomaterials, stem cells have better capacity to differentiate into ligament, fibrocartilage, and bone, as well as regulate inflammation through paracrine pathways, promoting graft regeneration. The application of stem cells in the clinic often results in disappointing outcomes and needs further investigations.

## Figures and Tables

**Table 1:** Recent animal studies on stem cell therapy for ACL graft regeneration.

	Author	Augmentation/induction	Animal	Evaluation	Outcome	Other outcomes
BMSCs	Hur	Fibrin glue	Rabbit	His, CT	+	
	Lim	Fibrin glue	Rabbit	His, Mech	+	
	Fan	Silk scaffold	Rabbit	His, Mech, CT	+	
	Fan	Silk scaffold	Pig	His, Mech, CT	+	
	Li	Triphasic silk graft	Rabbit	His, Mech, CT	+	
	Zhu	Electrospun scaffolds	Rabbit	His, Mech, CT	+	Lattice-like nanofibrous meshes enhance osteogenic differentiation
	Vaquette	PCL electrospun mesh	Sheep	His, Mech	+	
	Zhang	PLGA silk scaffold	Rabbit	His, Mech	+	
	Li	Cu-BG/PET	Rat	His, Mech, CT	+	
	Lu	Decellularized allogenic ST	Rabbit	His, Mech, CT	+	Decellularized allograft+BMSCs are better than allograft
	Setiawati	VEGF	Rabbit	His, Mech, MRI	+	
	Teng	PRP	Rabbit	His, Mech, CT	+	PRP enhances osteogenic differentiation
	Zhu	BMP2 gene therapy	Rabbit	His, Mech	+	
	Chen	bFGF/BMP2 gene therapy	Rabbit	His, Mech, CT	+	Combined BMP2 and bFGF exerted more potent effects than lone growth factor
	Wang	TGF gene therapy	Rabbit	His, Mech, CT	+	
	Dong	BMP2 gene therapy	Rabbit	His, Mech	+	
	Wei	TGF*β*/VEGF gene therapy	Rabbit	His, Mech	+	Combined TGF*β*-1 and VEGF165 exerted more potent effects than lone growth factor
	Li	PDGF gene therapy	Rabbit	His	+	
	Fan	Triphasic silk scaffold (TGF-*β*3 and BMP2 gene therapy)	Rabbit	His, Mech	+	
	Pauly	CTGF-electrospun scaffolds	Rabbit	His, X-ray	+	
ADSCs	Kosaka	Fibrin glue	Rabbit	His, Mech	+	
	Teuschl	Silk scaffold	Sheep	His, CT	(-)	
	Parry	PCLF+PET scaffold	Rabbit	His, Mech, CT	+	
	Kouroupis	Leeds-Keio biomaterial; BMP-2/FGF-2	Pig	His, Mech	/	BMP-2/FGF-2 induced stem cells to differentiate towards bone and ligament at the ends and central part of the biomaterial scaffold
	Zhang	Runx2 gene therapy	Rabbit	His, Mech, CT	+	Runx2 enhances osteoblast differentiation and inhibits adipogenic differentiation
LDSCs	Mifune	Injected	Rat	His, Mech, CT	+	
	Mifune	Cell sheet	Rat	His, Mech	+	Cell sheet is better than injection
	Ruan	Silk-collagen sponge scaffold	Rabbit	His, X-ray	+	
	Hu	SDF-1 releasing collagen-silk	Rabbit	His, CT	+	
	Takayama	VEGF gene therapy	Rat	His, Mech	/	CD34+ LDSCs have positive effects; overexpression of VEGF impairs biomechanics
	Kawakami	BMP2 gene therapy	Rat	His, Mech	+	BMP2 enhances osteogenic differentiation
TDSCs	Lui	Cell sheet	Rat	His, Mech, CT	+	
sMSCs	Ju	Gel injection	Rat	His	+	
hUCB-MSCs	Jang	Fibrin glue	Rabbit	His, CT	+	
	Park	3D bio-printed scaffold	Rabbit	His, CT	+	

PRP: platelet-rich plasma; His: histology; Mech: mechanics; PCLF+PET: polycaprolactone fumarate scaffolds with polyethylene terephthalate; bFGF: basic fibroblast growth factor; BMP2: bone morphogenetic protein 2; TGF: transforming growth factor; VEGF: vascular endothelial growth factor; PDGF: platelet-derived growth factor; ST: semitendinosis; PCL: polycaprolactone; BMSCs: bone marrow-derived mesenchymal stem cells; ADSCs, adipose-derived stem cells; sMSCs: synovial mesenchymal stem cells; hUCB-MSCs: human umbilical cord blood-derived mesenchymal stem cells; PLGA: lactic-co-glycolic acid; Cu-BG/PET: copper-containing bioactive glass polyethylene terephthalate; Runx2; PCLF+PET: polycaprolactone fumarate+polyethylene terephthalate sutures; SDF: stromal cell-derived factor 1; CTGF: connective tissue growth factor; bFGF: basic fibroblast growth factor.

**Table 2:** The advantages and disadvantages of commonly utilized stem cell types in ACL graft regeneration.

Formulation	Msc content	Advantages	Disadvantages
BMSCs	0.01-0.001% [142]	Great proliferation Low cost Low immunoreaction Easy to obtain	Low content Donor pain and infection Less homogeneous
ADSCs	~1% [143]	Abundant resource More homogeneous Factor secretion Less immunogenic than BMSCs	Enzymatic processing Low ligament differentiative potential [53]
TDSCs/LDSCs	3-4% [56]	Same derived resource Better epigenetic regulation [144] Cell-line maintainment [145]	Slow growth Low content
ESC	—	Indefinite self-renewal [146] Totipotency	Ethic issue Tumorigenicity [147] Immunogenicity [148]

BMSCs: bone marrow-derived mesenchymal stem cells; ADSCs: adipose tissue-derived stem cells; TDSCs/LDSCs: tendon/ligament stem/progenitor cells; ESCs: embryonic stem cells.

**Table 3:** Published clinical trials of stem cell therapy for ACL graft regeneration.

Author	Cell resource	Patient^∗^	Follow-up	Evaluation	Outcome	Other outcomes
Wang et al. [112]	BMSCs	11 vs. 6	2 y	Adverse event; pain; function; MRI; LifeQ	+	Less pain, symptoms, bone expansion, joint space narrowing, and cartilage volume loss
Silva et al. [149]	BMSCs	20 vs. 23	1 y	MRI	-	No signal-to-noise ratio difference
Alentorn-Geli et al. [115]	ADSCs	20 vs. 19	1 y	Pain; function; MRI	-	
Park et al. [116]	hUCB-MSCs	10 vs. 10 vs. 10^#^	2 y	Adverse event; KT; function; arthroscopy	—	Safe but no clinical advantage

^∗^The experimental group (ACLR+stem cell) vs. the negative control group (ACLR); ^#^the experimental group (ACLR+stem cell+HA) vs. the negative control group (ACLR) vs. the positive control group (ACLR+HA).

## Supplementary Materials

Supplementary Materials The detail of clinical trials in the application of different stem cell lineages after ACL reconstruction. Description: a comprehensive search of the literature was carried out in Sep. 2nd, 2020, using electronic databases PubMed, Embase, and Cochrane Library. The keywords “anterior cruciate ligament” and “stem cell” were combined without language or time restrictions. The results were limited to RCTs.
