# Supplementary material for: Application of Stem Cell Therapy for ACL Graft Regeneration
Source: Stem Cells Int. 2021 Aug 2;2021:6641818. doi: 10.1155/2021/6641818 (PMC8352687; doi:10.1155/2021/6641818)
Supplement: Supplementary Materials — The detail of clinical trials in the application of different stem cell lineages after ACL reconstruction. Description: a comprehensive search of the literature was carried out in Sep. 2nd, 2020, using electronic databases PubMed, Embase, and Cochrane Library. The keywords “anterior cruciate ligament” and “stem cell” were combined without language or time restrictions. The results were limited to RCTs. [file 6641818.f1.docx]

**Supplementary materials**

The detail of clinical trials are included in Figure 3. A comprehensive search of the literature was carried out in Sep. 2nd 2020 using electronic databases PubMed, Embase, and Cochrane library. The keywords “Anterior cruciate ligament” and “stem cell” were combined without language or time restrictions. The results were limited to RCTs.

# Characteristics of ongoing clinical trials of stem cells in ACLR (2020.09.02)

**NCT04205656**

| Title | Prospective Evaluation of PRP and BMC Treatment to Accelerate Healing After ACL Reconstruction |
| --- | --- |
| Identifier | NCT04205656 |
| Status (23th April 2020) | “This study is ongoing, and is currently recruiting participants.” |
| Methods | Randomized controlled trial |
| Participants | Inclusion criteria   - Age 16-50 - Acute ACL injury within 6 months - Scheduled for unilateral, primary ACLR with a BTB (bone-patellar tendon-bone) autograft; - The following concomitant injuries are allowed: Meniscal injuries; Articular chondral injury that can be addressed with debridement or chondroplasty; Tibial impaction fractures; Non-operative sprains/injuries of MCL or LCL;   Exclusion criteria   - pregnant; - Previous surgery for either knee except in cases of prior diagnostic arthroscopy and/or minimal debridement; - Significant osteoarthritis (OA) of the knee (e.g. grade 4 with cystic changes and/or significant osteophytes); - Concomitant cartilage restoration procedure - Biologic treatment in the operative knee within 6 months - Steroid injections in the operative knee within 3 months - ACLR which requires the following concomitant treatments: bone plating, metal implants (with the exception of titanium interference screws) or microfracture, etc. |
| Intervention | Bone Marrow Concentrate (BMC) stem cells |
| Comparator | ACL reconstruction (group 1), ACL reconstruction + PRP (group 2) |
| Registration date | December 2019 |
| Estimated completion date | December 2022 |
| Contact details | Holly del Junco. The Steadman Clinic  Vail, Colorado, United States, 81657 |

**ACTRN12609001018202**

| Title | A randomised, double-blind, comparative study to assess the safety & tolerability of a single injection into the knee joint of MSB-CAR001 when combined with Hyaluronan, as compared to Hyaluronan alone in patients who have recently undergone an Anterior Cruciate Ligament Reconstruction. |
| --- | --- |
| Identifier | ACTRN12609001018202 |
| Status (23th April 2020) | “This study is ongoing, and recruitment is completed.” |
| Methods | Randomized controlled trial |
| Participants | Inclusion criteria   - 18-40 yrs - Anterior cruciate ligament (ACL) injury requiring reconstruction within 6 months of initial ACL injury; - Have undergone unilateral ACL reconstruction surgery within 6 months of injury; - Clinically stable knee after reconstruction - autograft hamstring;   Exclusion criteria   - surgery and radiation therapy in recent 6 weeks - Pregnancy - combine ligament instability = grade II in physical examination (grade 0: none, grade I: 0~5mm, grade II: 5~10mm, grade III: >10mm) |
| Intervention | A single injection of Mesenchymal Precursor Cells MSB-CAR001, of 75 million cells per dose mixed with 2ml Hyaluronan and injected into the knee |
| Comparator | ACL reconstruction + hyaluronic acid |
| Registration date | 24/11/2009 |
| Estimated completion date | None stated. |
| Contact details | Mr Michael DiMuro  505 Fifth Avenue Level 3 New York, NY 10017 United States of America |

**NCT04178538**

| Title | The Effects of Fertilized ACL Technique on Outcomes of ACL Reconstruction in Young Adults (FACL) |
| --- | --- |
| Identifier | NCT04178538 |
| Status (23th April 2020) | “This study is ongoing, and is currently recruiting participants.” |
| Methods | Randomized controlled trial |
| Participants | Inclusion criteria   - Patients must be age 14-60 years old. - Must be skeletally mature (Tanner 4) patients, with an ACL deficient knee who desire to have ACL reconstructive surgery using autograft or allograft augmentation. - Patients with associated meniscal and chondral pathology (except patients falling into exclusion criteria below) will be included in the study; such pathology will be treated at the time of ACL reconstruction at the discretion of the surgeon, (and such pathology and treatment will be recorded). - An understanding of the purpose of the study, and have signed the informed consent. - Able to return for all subsequent study visits   Exclusion criteria   - Patients with multi-ligament surgery (MCL, PCL, LCL, PMC, or PLC repair or reconstruction), - Patients whom have had previous ACL reconstructive surgery on ipsilateral knee. - Patients who are currently pregnant or nursing. - Patients who have a current infection at the operative site. - Any condition or personal issue that the surgeon deems ineffective to the outcome of the study. - Workmen's compensation cases |
| Intervention | ACL reconstruction with bone marrow, demineralized bone marix, and internal brace augmentation |
| Comparator | Standard ACL reconstruction with all inside technique |
| Registration date | December 1, 2019 |
| Estimated completion date | September 11, 2020 |
| Contact details | Chad Lavender, MD  Marshall University Department of OrthopedicsHuntington, West Virginia, United States |

**NCT03294759**

| Title | Bio ACL Reconstruction Amnion Collagen Matrix Wrap and Stem Cells |
| --- | --- |
| Identifier | NCT03294759 |
| Status (23th April 2020) | “This study is ongoing, but not recruiting participants.” |
| Methods | Randomized controlled trial |
| Participants | Inclusion criteria   - Patients between the ages of 18 and 45 who are scheduled to have anterior cruciate ligament reconstruction with autologous grafts by one of the investigating physicians will be screened for participation in this study. - Patients must be willing to undergo MRI scans post -operatively at 3, 6, 9 months and 1 year   Exclusion criteria   - Patients with prior procedures or significant prior injuries to the same knee are excluded. - - Any patient who will have difficulty obtaining internet access, does not have an active e-mail address, or is unable to comprehend study documents or give informed consent will be excluded. - Patient who are unable to complete MRI examinations due to claustrophobia or anxiety |
| Intervention | ACL reconstruction. The graft will be wrapped in a collagen wrap and injected with aspirate from the patient's bone marrow. |
| Comparator | ACL reconstruction |
| Registration date | September 6, 2017 |
| Estimated completion date | February 25, 2021 |
| Contact details | Adam Anz, MD  Andrews Institute Gulf Breeze, Florida, United States, 32561 |

**NCT02469792**

| Title | Effectiveness and Safety of Autologous ADRC for Treatment of Anterior Cruciate Ligament Partial Rupture |
| --- | --- |
| Identifier | NCT02469792 |
| Status (23th April 2020) | “This study is ongoing.” |
| Methods | Single Group Assignment |
| Participants | Inclusion criteria   - partial rupture of anterior cruciate ligament (confirmed by MRI or knee arthroscopic surgery) - Clinically significant knee instability (positive anterior drawer test, pivot shift test, and Lachman test) - Patient is familiar with Participant information sheet - Patient signed informed consent form   Exclusion criteria   - Knee osteoarthritis grade III and grade IV - Medical history of autoimmune diseases - Patients prescribed for immunosuppressive treatment - Contraindications to the general or local anesthesia or medical history of allergic reactions to anesthetics - Subcompensated or decompensated forms of chronic diseases of internal organs, ect. |
| Intervention | Autologous adipose-derived regenerative cells (ADRC) |
| Comparator | none |
| Registration date | June 2015 |
| Estimated completion date | December 2018 |
| Contact details | Sergey V Ivannikov, Professor  Federal State Budgetary Institution "Central Clinical Hospital with Outpatient Health Center" of the Business Administration for the President of the Russian Federation; Center for Biomedical Technologies  Moscow, Russian Federation, 121359 |
